# 

**Published:** 1888-10

**Authors:** 


THE TRANSACTIONS OF THE TEXAS STATE MEDICAL ASSOCIATION, twentieth annual session, held at Galveston April last, is out, and presents a very creditable appearance. Compared with former editions, the present volume contains a less number of pages, but the matter is more select.

The amount of money at the disposal of the Publishing Committee with which to pay for the transactions, was limited, and, as explained in a note, the committee was compelled to omit the Constitution and By-Laws, together with a number of papers, which did not come up to the requirements. Several papers, however, were thrown into the waste-basket, owing to their defective composition, which act on the part of the Publishing Committee, is specially to be commended.

The President's retiring address should be read by everybody in the land.

The portrait of Dr. J. F. Y. Paine, the new President, reminds one of Victor Emanuel. B.

---

BOOK NOTICES.—Again we are compelled to omit Book Notices to make room for other matter. "Next time," etc.

☞ BIG INDUCEMENTS will be offered live, active canvassers to take hold of the subscription of the JOURNAL. Call on or address the editor. Physicians can easily make their own subscription clear by sending us two other names and four dollars.

